# Combined Effect of Hemostatic Gene Polymorphisms and the Risk of Myocardial Infarction in Patients with Advanced Coronary Atherosclerosis

**DOI:** 10.1371/journal.pone.0001523

**Published:** 2008-02-06

**Authors:** Nicola Martinelli, Elisabetta Trabetti, Mirko Pinotti, Oliviero Olivieri, Marco Sandri, Simonetta Friso, Francesca Pizzolo, Claudia Bozzini, Pier Paolo Caruso, Ugo Cavallari, Suzanne Cheng, Pier Franco Pignatti, Francesco Bernardi, Roberto Corrocher, Domenico Girelli

**Affiliations:** 1 Department of Clinical and Experimental Medicine, University of Verona, Verona, Italy; 2 Section of Biology and Genetics, Department of Mother and Child and Biology–Genetics, University of Verona, Verona, Italy; 3 Department of Biochemistry and Molecular Biology, University of Ferrara , Ferrara, Italy; 4 Department of Human Genetics, Roche Molecular Systems, Inc., Alameda, California, United States of America; Peninsula Medical School, United Kingdom

## Abstract

**Background:**

Relative little attention has been devoted until now to the combined effects of gene polymorphisms of the hemostatic pathway as risk factors for Myocardial Infarction (MI), the main thrombotic complication of Coronary Artery Disease (CAD). The aim of this study was to evaluate the combined effect of ten common prothrombotic polymorphisms as a determinant of MI.

**Methodology/Principal Findings:**

We studied a total of 804 subjects, 489 of whom with angiographically proven severe CAD, with or without MI (*n* = 307; *n* = 182; respectively). An additive model considering ten common polymorphisms [Prothrombin 20210G>A, PAI-1 4G/5G, Fibrinogen β -455G>A, FV Leiden and “R2”, FVII -402G>A and -323 del/ins, Platelet ADP Receptor P2Y12 -744T>C, Platelet Glycoproteins Ia (873G>A), and IIIa (1565T>C)] was tested. The prevalence of MI increased linearly with an increasing number of unfavorable alleles (χ^2^ for trend = 10.68; *P* = 0.001). In a multiple logistic regression model, the number of unfavorable alleles remained significantly associated with MI after adjustment for classical risk factors. As compared to subjects with 3-7 alleles, those with few (≤2) alleles had a decreased MI risk (OR 0.34, 95%CIs 0.13–0.93), while those with more (≥8) alleles had an increased MI risk (OR 2.49, 95%CIs 1.03–6.01). The number of procoagulant alleles correlated directly (r = 0.49, *P* = 0.006) with endogenous thrombin potential.

**Conclusions:**

The combination of prothrombotic polymorphisms may help to predict MI in patients with advanced CAD.

## Introduction

Myocardial Infarction (MI), the leading complication of coronary atherosclerotic disease (CAD), generally occurs in the late stages of disease because of coronary thrombosis superimposed on a ruptured/unstable plaque [1]. In clinical practice it is well-known that, in spite of the documented presence of advanced CAD, only a subset of patients develops acute MI during their life-course [2]. The reasons for individual differences in susceptibility to MI are poorly understood. In principle, subjects with an increased tendency to form blood clots (i.e. with “hypercoagulability”) may be at increased risk, as observed for venous thrombosis. Lessons from animal models suggest that excessive thrombin generation may be particularly harmful during the later stages of atherosclerosis, when thrombotic complications often occur [3], [4]. However, this is difficult to assess in clinical practice, since we lack a unique and reliable laboratory marker of hypercoagulability [5]. Moreover, functional tests evaluating concentration and/or function of blood coagulation proteins are often subjected to multiple transient interferences, e.g. due to the use of antithrombotic and anticoagulant agents or the presence of concomitant inflammation. Genetic polymorphisms with a documented functional effect on blood coagulation proteins may represent a useful tool, by reflecting the individual's lifelong exposure to even a mild prothrombotic state. During the last decade, extensive studies on various individual polymorphisms as risk factors for CAD and MI have yielded largely inconclusive results [6]–[11]. These results reflect at least two critical issues: 1) the multifactorial and multistep pathogenesis of CAD, involving many different biochemical pathways and intermediate phenotypes (e.g. hyperlipidemia, diabetes, hypertension), each in turn under the control of many different genes; 2) the enormous heterogeneity of investigations in terms of study design, typology of patients included, and clinical endpoints [10]. There has also been relatively little attention devoted to assess the combined effect of genes, which might be anticipated by analogy to the well-known additive effects of conventional risk factors. Generally, individual polymorphisms confer a marginal to moderate CAD risk that becomes evident only across many thousands of individuals, as was recently demonstrated by meta-analysis for Factor V 1691 G>A (Factor V Leiden), prothrombin 20210 G>A, and PAI-1 -675 4G/5G [8]. This renders such polymorphisms unhelpful in assessing individuals' risk clinically. On the other hand, the value of analyzing multiple alleles simultaneously for determining the risk is not well studied.

In this study we evaluated the combined effect of ten common genetic variants, with known modest effects on the hemostatic balance (listed in Table 1) [6], [12]–[21], in modulating the risk of development of MI. Because of the relatively late occurrence of MI in the natural history of CAD, we focused on a selected population of high risk patients with angiographically documented, advanced CAD. A thrombin generation assay was also used in a subset of patients to explore the propensity to form blood clot as a function of the number of hemostatic polymorphisms.

**Table 1:** Description of the haemostatic gene polymorphisms, analysed in this study, and their associated intermediate phenotype.

Polymorphism	Chromosome location of gene	Effects on intermediate phenotype
**FIBRINOGEN beta-chain –455 G>A [fibrinogen]**	4q28	-455 AA genotype associated with fibrinogen concentrations that are 10% higher than GG genotype ^12^
**Factor VII A1/A2 [coagulation factor VII]**	13q34	A2 associated with reduced factor VII concentrations ^13^
**Factor VII–402 G>A [coagulation factor VII]**	13q34	-402A associated with increased factor VII concentrations ^14^
**Factor V Leiden (R506Q) [coagulation factor V]**	1q23	506Q is a cause of activated protein C (APC) resistance ^15^
**Factor V R2 (6755 A>G) [coagulation factor V]**	1q23	6755G associated with mild APC-resistance and impaired APC mediated factor VIII inactivation ^16^
**Prothrombin 20210 G>A [precursor of thrombin]**	11p11-q12	20210A associated with increased plasma prothrombin levels ^17^
**PAI-1–675 5G/4G [inhibitor of plasminogen activator]**	7q21.3-q22.1	4G associated with increased plasma PAI-1 Levels ^18^
**GP IIIa Leu33Pro [platelet receptor for fibrinogen and von Willebrand factor]**	17q21.32	33Pro might increase sensitivity to platelet aggregation ^19^
**GP Ia/IIa alfa2 873 G>A [platelet receptor for collagen]**	5q23-q31	873A (in linkage with 807T polymorphism) might increase the receptor density ^20^
**P2RY12 H1/H2 (-744T>C) [platelet receptor for ADP]**	3q24-q25	-744C, in absolute linkage disequilibrium with 3 others SNPs, marks the H2 haplotype, that is associated with maximal aggregation response to ADP ^21^

## Results

### Haemostatic polymorphisms in the CAD group as a whole versus CAD-free subjects

**Figure 1:**
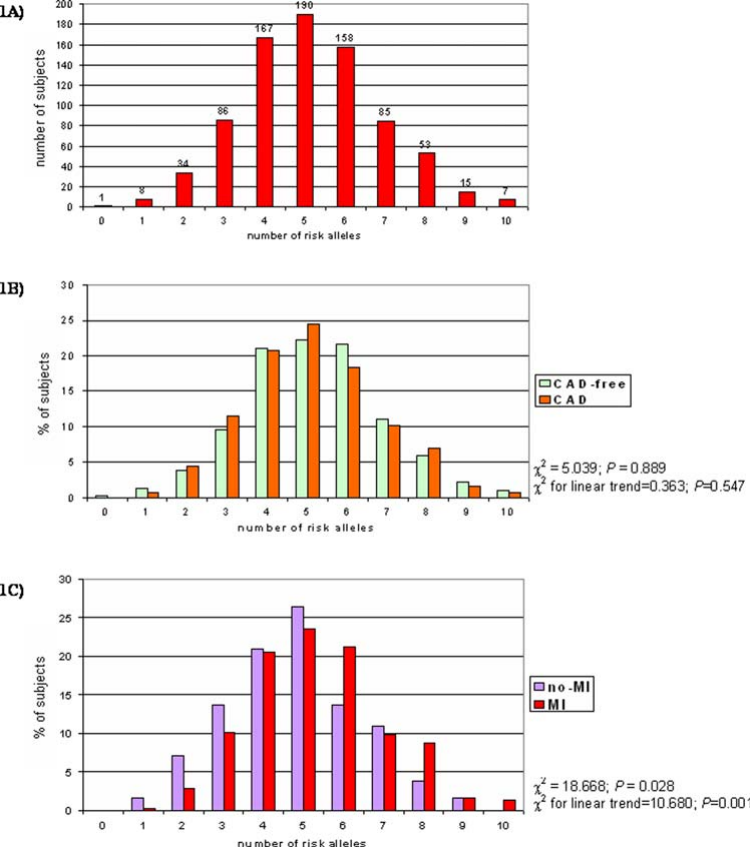
Study population (n = 804) stratified on the basis of number of risk alleles (1A). The distribution of number of risk alleles in CAD-free (n = 315) and in CAD patients (n = 489) (1B) and in CAD patients with (n = 307) or without a history of MI (n = 182) (1C).

Supplemental Table 1 (Table S1) shows the genotype frequencies for each of the 10 polymorphisms in CAD-free (n = 315; males 66.0%; mean age 59.2±11.9 years) and in CAD subjects (n = 489; males 83.6%; mean age 60.3±9.3 years). All alleles were in Hardy-Weinberg equilibrium. For each polymorphism there was no significant difference in genotype distribution between CAD and CAD-free groups. The distribution of the “prothrombotic score” (PS) in the whole study population (n = 804) is shown in figure 1A. The score ranged from 0 (1 subject) to 10 prothrombotic alleles (7 subjects), with a median level of 5. Figure 1B shows the distribution of the PS in CAD-free and in CAD subjects. No association was found between the PS and CAD (P = 0.889 by χ^2^-test).

### Individual haemostatic polymorphisms and MI risk in subjects with advanced CAD

Supplemental Table 2 (Table S2) shows the general characteristics of the CAD population divided in two groups on the basis of presence/absence of MI. As compared to CAD patients without MI, MI patients were significantly younger, more frequently males, had a higher degree of CAD in terms of number of diseased vessels, and lower HDL-cholesterol levels. No significant difference was found for the other variables. Table 2 shows the genotype frequencies of the 10 genetic variants in CAD patients with or without MI. Two polymorphisms, factor VII -402 G>A and fibrinogen β-chain -455 G>A, showed nominal association with MI at the univariate analysis. However, these associations were no longer significant after multiple logistic regression adjusted for sex, age, disease severity, smoking status, BMI, LDL- and HDL-cholesterol (P = 0.155 for factor VII -402 G/A and P = 0.998 for fibrinogen β-chain -455 G/A).

**Table 2:** Genotypes frequencies (%) of the CAD population, with or without MI.

	No MI (n = 182)	MI (n = 307)	*P* *
**FIBRINOGEN beta-chain –455 G>A**			
**GG**	65.4	59.9	0.028
**GA**	34.1	35.2	
**AA**	0.5	4.9	
**Factor VII A1/A2**			
**A1A1**	63.7	70.4	0.240
**A1A2**	31.3	26.7	
**A2A2**	5.0	2.9	
**Factor VII–402 G>A**			
**GG**	73.1	61.2	0.016
**GA**	23.1	35.5	
**AA**	3.8	3.3	
**Factor V Leiden (R506Q)**			
**RR**	96.7	96.8	0.725
**RQ**	3.3	2.9	
**QQ**	0	0.3	
**Factor V R2 (6755 A>G)**			
**AA**	83.5	82.1	0.917
**AG**	15.4	16.6	
**GG**	1.1	1.3	
**Prothrombin 20210 G>A**			
**GG**	96.7	94.1	0.204
**GA**	3.3	5.9	
**AA**	0	0	
**PAI-1–675 5G/4G**			
**4G-4G**	29.2	30.0	0.951
**4G-5G**	51.6	50.1	
**5G-5G**	19.2	19.9	
**GP IIIa Leu33Pro**			
**Leu/Leu**	75.8	69.7	0.210
**Leu/Pro**	23.7	28.3	
**Pro/Pro**	0.5	2.0	
**GP Ia/IIa alfa2 873 G>A**			
**GG**	41.2	37.8	0.740
**GA**	46.2	48.2	
**AA**	12.6	14.0	
**P2RY12 H1/H2 (-744T>C)**			
**TT**	77.5	73.6	0.234
**TC**	22.5	25.1	
**CC**	0	1.3	

* : by χ^2^-test

### Combined effect of haemostatic polymorphisms and MI risk

**Figure 2:**
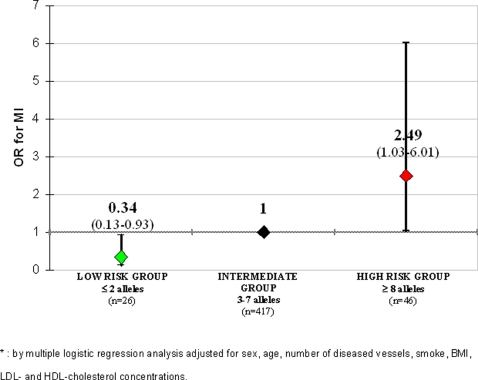
OR for MI in groups stratified on the basis of number of unfavourable alleles. The intermediate group (from 3 to 7 unfavourable prothrombotic alleles), representing the 85.3% of the whole population, is considered as reference group.

No significant interaction was found by CART among polymorphisms in determining MI risk (all P for interaction >0.05). As shown in figure 1C, the proportion of CAD patients with MI increased progressively with increasing number of unfavourable alleles (χ^2^ for linear trend = 10.68; P = 0.001). In a multiple logistic regression model the prothrombotic score remained significantly associated with MI after adjustment for sex, age, degree of CAD, smoke, BMI, LDL- and HDL-cholesterol (OR for 1-point increase in prothrombotic score = 1.22 with 95%CI 1.06–1.39, P = 0.004). Using the median of PS as cut-off, CAD patients with >5 alleles had a significantly increased risk of MI as compared to subjects with ≤5 alleles (OR 2.02 with 95%CI 1.27–3.21, P = 0.003, by multiple logistic regression). Using approximately the 5^th^ and the 95^th^ percentiles of PS distribution (i.e. 2 and 8, respectively), the study population could be classified into in 3 subgroups: a low-risk group with less than 3 unfavourable alleles (n = 26), an intermediate-risk group with 3 to 7 unfavourable alleles (n = 417), and a high-risk group with more than 7 unfavourable alleles (n = 46). The prevalence of MI among these groups increased progressively (38.5% in low-risk; 62.6% in intermediate-risk; and 78.3% in high-risk; P = 0.001 by χ^2^ for linear trend), while they were similar for the other clinical and laboratory variables (data not shown). Considering the intermediate-risk group as the reference group, carriers of <3 alleles had a lower risk of MI, while carriers of >7 alleles had an increased risk (Figure 2). Comparing the two extreme groups, the subjects with >7 alleles had a remarkably higher MI risk (OR 7.28 with 95%CI 2.01–26.36, P = 0.002 adjusted by multiple logistic regression). The ROC curve for information provided by our polygenic approach for MI prediction in CAD patients is plotted in Supplemental Figure 1 (Figure S1). The AUC was 0.581 with a 95% CI from 0.530 to 0.632.

### Combined effect of haemostatic polymorphisms and thrombin generation activity

**Figure 3:**
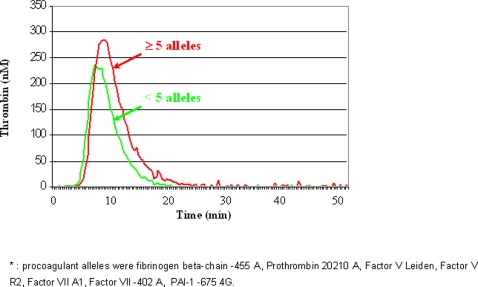
Comparison between the median thrombin generation activity curves in groups stratified on the basis of number of procoagulant alleles, with a threshold level at 5 alleles.

To get insights on the pathophysiological effect of combined hemostatic alleles, we assessed the characteristics of thrombin generation activity curves as a function of the number of procoagulant alleles (i.e. fibrinogen β-chain -455 A, Prothrombin 20210 A, Factor V Leiden, Factor V R2, Factor VII A1, Factor VII -402 A and PAI-1 -675 4G). Since this assay pertains only to the coagulation pathway, the three platelet-related polymorphisms were not considered for this analysis. This assay was performed in a subset of 29 CAD patients (26 males and 3 females, 22 with and 7 without MI), selected among those without possible confounders (i.e. concomitant anticoagulant therapy or overt signs of inflammation, documented by hs-CRP<5 mg/l), in order to form three groups matched for age and sex representing the previously defined risk groups (low-risk: n = 9, 8 males and 1 female, mean age 53.7±8.5; intermediate-risk: n = 10, 9 males and 1 female, mean age 57.8±7.4; high-risk: n = 10, 9 males and 1 female, mean age 56.0±8.6). The number of procoagulant alleles was significantly associated with ETP and with Start Tail, but not with Lag Time, Peak or Time to Peak (Table 3). Similarly, subjects with a high number of procoagulant alleles (≥5) had significantly higher ETP values as compared to subjects with fewer alleles (Table 4). These two groups were similar not only for age and sex, but also for smoking, hypertension and diabetes (data not shown). Their median thrombin generation activity curves are showed in Figure 3.

**Table 3:** Correlations between number of procoagulant alleles and different characteristics of thrombin generation activity.

	Pearson correlation coefficient	*P*
**Number of procoagulant alleles–ETP (nM×min)**	0.494	0.006
**Number of procoagulant alleles–Lagtime (min)**	0.187	0.332
**Number of procoagulant alleles–Peak (nM)**	0.244	0.203
**Number of procoagulant alleles–Time to Peak (min)**	0.230	0.230
**Number of procoagulant alleles–Start Tail (min)**	0.396	0.033

The analysis was performed in a subgroup of CAD patients (n = 29) without anticoagulant therapies and without signs of overt inflammation. Procoagulant alleles were fibrinogen beta-chain -455 A, Prothrombin 20210 A, Factor V Leiden, Factor V R2, Factor VII A1, Factor VII -402 A, PAI-1 -675 4G.

**Table 4:** Characteristics of thrombin activity generation curves in groups stratified on the basis of number of procoagulant alleles, with a threshold level at 5 alleles.

	<5 alleles (n = 19)	≥5 alleles (n = 10)	*P* †
**ETP (nM×min)**	1,341±158	1,661±277	0.005
**Lagtime (min)**	6.49±0.79	6.89±1.33	0.326
**Peak (nM)**	191±37	221±43	0.058
**Time to Peak (min)**	10.08±0.87	10.64±1.59	0.224
**Start Tail (min)**	27.76±2.84	30.20±3.40	0.050

The analysis was performed in a subgroup of CAD patients (n = 29) without anticoagulant therapies and without signs of overt inflammation. Procoagulant alleles were fibrinogen beta-chain -455 A, Prothrombin 20210 A, Factor V Leiden, Factor V R2, Factor VII A1, Factor VII -402 A, PAI-1 -675 4G.

† : by t-test

## Discussion

Evidence that a hypercoagulable state is associated with increased mortality has been provided by some recent studies [11], [22]. To our knowledge, this is the first study that attempts to look at the impact of the combined effect of several common prothrombotic polymorphisms in the identification of CAD patients at different risk of developing MI. To put our results into perspective, we propose the following considerations.

### Single haemostatic polymorphisms and MI risk

This study focused on relatively few genetic variants associated with defined biochemical alterations. While some of them (i.e. Factor V Leiden and prothrombin 20210 G>A) are established risk factors for venous thromboembolism, their association with arterial thrombosis is much less convincing [6], [9]. Here too, despite some nominal significant *P* values, we found no consistent association when each polymorphism was considered individually. Indeed, CAD and MI are paradigms of complex disease, in which the effect of individual genes on the risk is anticipated to be weak [23], [24]. Moreover, emphasizing the principle that “the highest the allele effect, the lowest the allele frequency” [25], it is plausible that genetic variants such those investigated in the present study, relatively frequent in the general population, could have at best only a mild effect on a potentially lethal phenotype like MI. Indeed, until now only a recent large meta-analysis including tens of thousands of patients has been able to detect a moderate but significant increase in the risk of coronary disease associated with either the Factor V Leiden mutation or the prothrombin 20210A variant [8].

### Combined effect of haemostatic polymorphisms and MI risk

Recently, a polygenic approach has been demonstrated to be a valid tool to identify subjects at risk for another complex trait such as type 2 diabetes [26]. A similar strategy was used in the present study, suggesting that in subjects with advanced CAD, an increasing number of prothrombotic alleles may confer a significant risk of developing MI. It is biologically plausible that the simultaneous presence of several genetic variations with modest but defined effects on the hemostatic process could influence the risk of the major thrombotic complication in a given CAD patient. Under certain stimuli, such as plaque erosion or rupture, this condition may predispose to sustained thrombin generation leading to the acute thrombotic event [2]. Accordingly, our *in vitro* functional studies showed an association between the number of procoagulant alleles and thrombin generation. The latter is known to be a highly variable and complex phenomenon modulated by the interplay of several factors, none of them with predominant influence, many of them under genetic control [27]. It is noteworthy that our clinical model focused on a homogeneous group of patients with angiographically proven advanced CAD. Elegant studies in animal models, i.e. Factor V Leiden mice crossbred with apolipoprotein E–deficient mice, indicates that unregulated thrombin generation is particularly harmful during the later stages of atherosclerosis. [3], [4]. Conversely, a mild hypercoagulable state may be less meaningful in absence of underlying vulnerable atherosclerotic plaques. Our results may thus apply only to the specific clinical model of this study, and not to all CAD patients. While it is reasonable that genetically-induced excessive thrombin generation may be clinically relevant in subjects with extensive coronary plaques, this excess might be less influential in the atherogenetic process, where other genetic factors (i.e. those involved in modulation of lipid metabolism, antioxidant balance, and so on) may be prominent. This could explain why we found no association between the hemostatic polymorphisms and the CAD phenotype.

### Study limitations and strengths

One strength of our study is the clear definition of phenotypes, allowing comparison of patients with angiographically proven, advanced CAD, with or without MI. The CAD population had a substantial burden of traditional risk factors and thus represented a typical patient population seen in clinical practice.

Our study has several limitations, including the relatively low number of subjects and polymorphisms and a retrospective case-control design. In this setting the possible confounding of the survivor effect should also be taken into account. The prothrombotic score, calculated as the sum of prothrombotic alleles, is likely an oversimplification, since it standardized the contribution of each gene variant and does not allow distinguishing the possible different transmission models, as well as the different biological weight of the polymorphisms. Nevertheless, for complex traits the presence of additive effects of many genes is considered more likely than interactive effects [28], [29], and additive models have been shown to perform well, even when the underlying model is unknown [30], [31].

This study can be viewed as hypothesis-generating, shedding light into the potential usefulness of a polygenic approach in appropriate clinical contexts. Indeed, the predictive power of our additive genetic model was relatively low, yielding an area under the ROC curve (AUC) of 0.58. The AUC is a measure of the discriminatory power of a test, ranging from 0.5 for no discriminatory power to 1 for a perfect test [32]. We tested only ten polymorphisms here, and there is reason to believe that the predictive power of genetic information could be greater. Increasing technological resources with decreasing costs are likely to allow the inclusion, in models similar to that used in the present study, of other genetic variants reproducibly associated to functional consequences on coagulation factors, either newly identified (i.e. the long-anticipated genetic modulators of Factor VIII), or not included in this study (i.e. Factor XIII Val34Leu). With larger data sets, it may also be possible to capture gene-gene and gene-environmental factors.

### Conclusions

Our data support the idea that, while individual genetic susceptibility variants are of limited clinical use, the combined information from a number of these variants can permit the identification of groups of people at high and low risk of developing a complex trait such as MI [33], [34]. The polygenic model used in this study, considering the cumulative effect of hemostatic gene variants, was significantly associated to some *in vitro* measurements of thrombin generation. In the specific context of advanced CAD, similar approaches may be useful as surrogate markers of the propensity to form blood clots leading to MI. Further studies on larger samples are needed to confirm this intriguing working-hypothesis, as well as to improve predictive modelling.

## Materials and Methods

### Study population

This study was performed within the framework of the Verona Heart Project, a regional survey aimed to search for new risk factors for CAD and MI in subjects with objective angiographic documentation of their coronary vessels. Details about enrolment criteria have been described in detail elsewhere [35], [36]. A total of 804 subjects, for whom complete analyses of 10 polymorphisms of genes involved in hemostatic pathways were available, were included in the present study. Three-hundred fifteen subjects had completely normal coronary arteries, being submitted to coronary angiography for reasons other than CAD, mainly valvular heart disease (CAD-free group). These controls were also required to have neither history nor clinical or instrumental evidence of atherosclerosis in vascular districts beyond the coronary bed. Four-hundred eighty nine subjects had angiographically proven CAD (the majority of them being candidates for coronary artery bypass grafting) with objective documentation of presence/absence of MI. The disease severity was determined by counting the number of major epicardial coronary arteries (left anterior descending, circumflex, and right) affected with ≥1 significant stenosis (≥50%). According to the hypothesis to be tested, subjects with non-advanced CAD (i.e. coronary stenosis <50%) were not included in the study. Cardiologists unaware that the patients were to be included in the study assessed the angiograms. Patients were classified into MI (n = 307) and non-MI (n = 182) subgroups on the basis of a thorough review of medical records including history, electrocardiogram, enzyme changes, and/or the typical sequelae of MI on ventricular angiography.

All participants came from the same geographical area (Northern Italy), with a similar socio-economic background. At enrolment, a complete clinical history was collected, including the assessment of cardiovascular risk factors such as obesity, smoking, hypertension and diabetes. The study was approved by the Ethic Committee of our Institution (Azienda Ospedaliera, Verona). A written informed consent was obtained from all the participants after a full explanation of the study.

### Biochemical analysis

Samples of venous blood were drawn from each subject at enrolment, before coronary angiography and after an overnight fast. Serum lipids, as well as other CAD risk factors, including high-sensitivity C-reactive protein (hs-CRP) were determined as previously described [26].

### Genetic analysis and nomenclature

Genomic DNA was extracted from whole blood samples by a phenol-chloroform procedure using the Puregene kit (Gentra Systems) according to the manufacturer's protocol. The 10 genetic polymorphisms, selected on the basis of prior evidence of potential functionality in modulating the hemostatic pathway, are listed in Table 1. Seven out of ten polymorphisms (fibrinogen beta-chain -455G>A, Factor VII A1/A2, Factor V Leiden, Prothrombin 20210 G>A, PAI-1 -675 5G/4G, GP IIIa Leu33Pro, GP Ia/IIa alfa2 873 G>A) were examined by a previously described and validated linear-array assays for candidate markers [37]. The accuracy of the linear-array genotyping system as compared with standard genotyping approaches reported elsewhere [35], [36] was evaluated and the findings provide reassurance regarding the validity of the system used, as previously described [38]. The remaining three (Factor VII -402 G>A, Factor V R2, P2RY12 H1/H2) were analyzed by previously described, standard genotyping approaches [36], [39], [40]. Genotype interpretation for each polymorphism was performed independently by two investigators and very few samples (<1%) with unclear result were re-genotyped.

### Measurement of thrombin generation activity

This assay was performed in a subset of CAD patients on samples drawn at enrolment, in order to evaluate a possible functional counterpart of an increasing number of prothrombotic alleles in terms of propensity to form blood clot. Plasma sample were centrifuged at 23,000 g at 4°C for 1 hour before testing. Calibrated automated thrombin activity measurement was conducted according to Hemker et al. [41], [42] in a microtiter plate fluorometer (Fluoroskan Ascent, ThermoLabsystems, Helsinki, Finland) using the Thrombinoscope software (Synapse BV, Maastricht, The Netherlands). The assay was carried out at 37 °C essentially as previously reported [43]. Coagulation was triggered in platelet poor plasma by recalcification in the presence of 1 pM recombinant human tissue factor and 4 μM phospholipids. Thrombin generation was then evaluated overtime by exploiting a specific fluorogenic substrate (Z-Gly-Gly-Arg-AMC). Thrombin generation measurement was conducted in parallel in plasma samples after the addition of a thrombin calibrator provided by the manufacturer (Synapse BV). The software enables the estimate of the following parameters: a) the Lag Time of thrombin generation, b) the time to reach the maximum concentration of thrombin (time to Peak), c) the maximum concentration of thrombin (Peak), d) the total duration of thrombin generation activity (Start Tail), and e) the total amount of thrombin activity assessed as the area under the curve, i.e. the endogenous thrombin potential (ETP). All experiments were carried out in duplicate.

### Statistics

Calculations were performed mainly with SPSS 13.0 statistical package (SPSS Inc., Chicago, IL). Distributions of continuous variables in groups were expressed as means±standard deviation. Logarithmic transformation was performed on skewed variables, for whom geometric mean with 95% confidence interval (CI) are given. Quantitative data were assessed using the Student's t-test or by ANOVA with Tukey's post-hoc comparison of the means. Correlations between quantitative variables were assessed using Pearson's correlation test. Qualitative data were analyzed with the χ^2^-test or the Fisher exact-test when indicated. Hardy-Weinberg equilibrium was tested for each genotype within each group by means of χ^2^-test. A value of P<0.05 was considered statistically significant.

Within each group examined, the frequencies of the genotypes associated with each of the polymorphisms were compared by the χ^2^-test, with the values predicted on the basis of the Hardy-Weinberg equilibrium. To assess the extent to which gene polymorphisms were associated with MI, odds ratios with 95% CIs were estimated by univariate logistic regression analysis. Adjustment for other variables (i.e. number of affected vessels, age, sex, smoking, BMI, LDL- and HDL-cholesterol) was performed by adding those covariates in a set of multiple logistic-regression models.

The existence of gene-gene interactions was first explored by a data mining technique similar to the Adaboost algorithm, and based on classification and regression trees (CART): the gradient boosting machine [44]. The statistical significance of the interactions found with this method was then estimated by the likelihood ratio test applied on two logistic models (with and without the interaction terms). After observing that no significant interaction was present, we focused on an additive model. On this basis, we attributed to each patient a “prothrombotic score” (PS), reflecting the sum of 10 concomitant unfavourable prothrombotic alleles, theoretically ranging from 0 (no prothrombotic allele present) to 20 (all the prothrombotic alleles present). The association between the prothrombotic score and MI was evaluated by χ^2^ for linear trend analysis. The prothrombotic score was analysed by logistic regression both as continuous variable and as categorised variable. Odds ratios with 95% CIs were estimated by univariate logistic regression analysis and then by multiple logistic regression with adjustment for number of affected vessels, age, sex, smoking, BMI, LDL- and HDL-cholesterol. The predictivity of our models was then evaluated by the receiver-operating-characteristics (ROC) curve, estmating the area under the curve (AUC).

## Supporting Information

Table S1 Genotypes frequencies (%) in CAD-free and in CAD subjects.
(0.08 MB DOC)

Table S2 Characteristics of the CAD population, with or without MI.
(0.06 MB DOC)

Figure S1 ROC for the information provided by our polygenic model of prothrombotic alleles after fitting a logistic regression model.
(0.05 MB DOC)
