# Supplementary material for: Combined Effect of Hemostatic Gene Polymorphisms and the Risk of Myocardial Infarction in Patients with Advanced Coronary Atherosclerosis
Source: PLoS One. 2008 Feb 6;3(2):e1523. doi: 10.1371/journal.pone.0001523 (PMC2211406; doi:10.1371/journal.pone.0001523)
Supplement: Table S1 — Genotypes frequencies (%) in CAD-free and in CAD subjects. (0.08 MB DOC) [file pone.0001523.s001.doc]

**Table S1:** Genotypes frequencies (%) in CAD-free and in CAD subjects.

|  | **CAD-free**  **(n=315)** | **CAD**  **(n=489)** |
| --- | --- | --- |
| **FIBRINOGEN beta-chain –455 G>A** |  |  |
| **GG** | 61.6 | 62.0 |
| **GA** | 32.1 | 34.8 |
| **AA** | 6.3 | 3.3 |
| **Factor VII A1/A2** |  |  |
| **A1A1** | 70.5 | 67.9 |
| **A1A2** | 25.1 | 28.4 |
| **A2A2** | 4.4 | 3.7 |
| **Factor VII – 402 G>A** |  |  |
| **GG** | 64.1 | 65.6 |
| **GA** | 32.7 | 30.9 |
| **AA** | 3.2 | 3.5 |
| **Factor V Leiden (R506Q)** |  |  |
| **RR** | 92.7 | 96.7 |
| **RQ** | 7.3 | 3.1 |
| **QQ** | 0 | 0.2 |
| **Factor V R2 (6755 A>G)** |  |  |
| **AA** | 87.9 | 82.6 |
| **AG** | 10.8 | 16.2 |
| **GG** | 1.3 | 1.2 |
| **Prothrombin 20210 G>A** |  |  |
| **GG** | 94.6 | 95.1 |
| **GA** | 5.1 | 4.9 |
| **AA** | 0.3 | 0 |
| **PAI-1 – 675 5G/4G** |  |  |
| **4G-4G** | 31.4 | 29.7 |
| **4G-5G** | 50.8 | 50.7 |
| **5G-5G** | 17.8 | 19.6 |
| **GP IIIa Leu33Pro** |  |  |
| **Leu/Leu** | 70.8 | 72.0 |
| **Leu/Pro** | 26.3 | 26.6 |
| **Pro/Pro** | 2.9 | 1.4 |
| **GP Ia/IIa alfa2 873 G>A** |  |  |
| **GG** | 40.3 | 39.1 |
| **GA** | 46.7 | 47.4 |
| **AA** | 13.0 | 13.5 |
| **P2RY12 H1/H2 (-744T>C)** |  |  |
| **TT** | 79.0 | 75.1 |
| **TC** | 19.1 | 24.1 |
| **CC** | 1.9 | 0.8 |
