# Supplementary material for: Combined Effect of Hemostatic Gene Polymorphisms and the Risk of Myocardial Infarction in Patients with Advanced Coronary Atherosclerosis
Source: PLoS One. 2008 Feb 6;3(2):e1523. doi: 10.1371/journal.pone.0001523 (PMC2211406; doi:10.1371/journal.pone.0001523)
Supplement: Table S2 — Characteristics of the CAD population, with or without MI. (0.06 MB DOC) [file pone.0001523.s002.doc]

**Table S2:** Characteristics of the CAD population, with or without MI.

| **Characteristics** | **No MI**  **(n=182)** | **MI**  **(n=307)** | ***P*** |
| --- | --- | --- | --- |
| **Age (years)** | 61.9 ± 7.8 | 59.4 ± 9.9 | 0.004 * |
| **Male sex (%)** | 76.9 | 87.6 | 0.002 ^#^ |
| **BMI (kg/m²)** | 27.1 ± 3.4 | 26.5 ± 3.2 | 0.093 * |
| **Hypertension (%)** | 62.8 | 59.9 | 0.536 ^#^ |
| **Smoking (%)** | 64.2 | 71.6 | 0.094 ^#^ |
| **Diabetes (%)** | 18.3 | 14.0 | 0.200 ^#^ |
| **estimated GFR (ml/min) ^** | 72.4 ± 16.0 | 72.0 ± 16.5 | 0.798 * |
| **Total cholesterol (mmol/L)** | 5.82 ± 1.10 | 5.91 ± 1.14 | 0.399 * |
| **LDL-cholesterol (mmol/L)** | 3.86 ± 1.01 | 4.02 ± 0.97 | 0.116 * |
| **HDL-cholesterol (mmol/L)** | 1.28 ± 0.35 | 1.18 ± 0.29 | 0.002 * |
| **Triglycerides (mmol/L)** | 1.98 ± 1.16 | 2.03 ± 1.16 | 0.676 * |
| **hs-CRP (mg/L)** | 3.09 (2.60-3.68) | 2.97 (2.61-3.37) | 0.707 * |
| **Number of vessel involved (%)** |  |  | < 0.001^#^ |
| **1 vessel** | 23.8 | 7.3 |
| **2 vessels** | 14.9 | 21.2 |
| **3 vessels** | 60.8 | 70.5 |
| **Left main coronary artery** | 0.6 | 1.0 |

* : by t-test

# : by χ² test

^ : glomerular filtration rate (GFR) estimated by the abbreviated Modification of Diet in Renal Disease (**MDRD**) equation.
