# Supplementary material for: Combined Effect of Hemostatic Gene Polymorphisms and the Risk of Myocardial Infarction in Patients with Advanced Coronary Atherosclerosis
Source: PLoS One. 2008 Feb 6;3(2):e1523. doi: 10.1371/journal.pone.0001523 (PMC2211406; doi:10.1371/journal.pone.0001523)
Supplement: Figure S1 — ROC for the information provided by our polygenic model of prothrombotic alleles after fitting a logistic regression model. (0.05 MB DOC) [file pone.0001523.s003.doc]

**Figure S1:** ROC for the information provided by our polygenic model of prothrombotic alleles after fitting a logistic regression model.

Area under the ROC curve with 95%CI:

**0.581 (0.530-0.632)**
